# Supplementary material for: Seasonal Trend of Viral Prevalence and Incidence of Febrile Convulsion: A Korea Public Health Data Analysis
Source: Children (Basel). 2023 Mar 9;10(3):529. doi: 10.3390/children10030529 (PMC10047246; doi:10.3390/children10030529)

Table S1: Positive detection rates of virus during study period.

Mon PDR(%)	Jan	Feb	Mar	Apr	May	Jun	Jul	Aug	Sep	Oct	Nov	Dec
						**HAdV**						
2015	5.04	2.15	2.83	4.70	4.83	5.63	3.32	3.18	2.30	6.98	9.03	8.78
2016	9.18	3.88	3.93	5.54	6.53	6.95	7.16	9.48	9.58	6.50	5.83	3.24
2017	3.72	2.53	2.43	2.74	6.05	4.73	2.96	5.18	4.33	3.78	4.73	2.60
2018	2.98	2.80	4.25	5.38	5.45	6.80	5.82	9.55	13.33	11.30	10.70	7.63
2019	6.68	5.05	4.85	4.43	7.84	10.40	7.52	12.88	10.20	9.44	9.75	8.83
						**HPIV**						
2015	0.92	0.55	0.78	6.30	17.65	11.75	7.04	6.28	8.93	4.72	9.53	4.08
2016	2.24	1.30	1.40	5.60	16.55	16.23	9.68	10.25	8.68	4.56	1.95	0.78
2017	1.58	1.70	2.98	8.38	19.43	12.83	9.68	8.95	4.60	3.04	3.33	2.68
2018	0.84	1.60	3.25	9.44	19.10	16.78	11.12	7.75	4.33	4.00	1.75	1.03
2019	1.06	1.10	2.33	3.48	13.56	20.35	15.74	9.70	7.58	3.98	2.55	1.65
						**HRSV**						
2015	8.70	2.15	1.55	0.40	0.58	0.45	0.12	0.13	0.00	0.96	4.13	14.40
2016	8.56	1.00	1.43	0.56	0.70	0.15	0.34	0.28	1.78	6.46	17.83	13.02
2017	5.16	3.83	2.53	0.64	0.10	0.23	0.60	0.85	3.30	5.24	17.25	10.73
2018	4.58	4.25	3.65	0.64	0.50	0.40	0.72	1.05	1.55	4.52	13.95	11.20
2019	6.40	3.80	1.65	0.70	0.44	0.10	0.10	0.85	1.68	3.72	10.10	11.73
						**IFV**						
2015	22.80	48.85	45.28	25.96	3.10	0.33	0.14	0.00	0.38	0.10	0.48	1.53
2016	15.24	47.55	36.95	26.60	5.00	0.70	0.22	0.28	0.25	0.40	0.80	35.50
2017	26.20	7.70	8.95	12.72	5.03	2.20	1.10	0.55	0.93	0.66	4.05	41.55
2018	58.50	35.63	10.55	3.46	2.80	1.08	0.50	0.30	0.58	1.78	11.15	35.35
2019	28.20	9.13	20.80	34.85	10.52	2.10	0.40	0.35	0.63	3.44	10.08	23.95
						**HCoV**						
2015	8.74	1.40	0.95	0.56	0.10	0.50	0.58	0.60	0.38	1.38	1.80	5.53
2016	7.96	3.98	4.95	4.26	4.90	3.08	3.94	4.53	4.68	4.24	7.80	9.82
2017	10.26	6.45	5.15	3.62	2.68	2.35	1.02	1.45	1.55	2.14	5.15	7.20
2018	6.76	9.88	7.73	3.52	0.85	0.48	1.00	3.13	2.60	5.80	10.93	9.83
2019	5.48	3.70	2.83	1.38	0.92	1.68	0.64	0.45	1.05	1.94	3.35	7.55
						**HRV**						
2015	8.80	7.73	10.08	15.84	17.10	19.30	21.62	18.10	25.40	28.82	29.03	16.78
2016	8.52	3.78	12.28	15.44	19.55	16.38	19.62	19.60	18.40	24.56	20.68	8.02
2017	9.56	15.40	20.90	18.66	14.78	17.78	21.40	30.63	33.10	24.68	23.88	6.83
2018	2.52	5.95	20.63	28.34	21.53	20.18	21.14	12.70	24.63	22.08	15.70	9.78
2019	8.68	14.33	18.85	16.43	19.82	17.45	23.00	13.45	21.48	22.62	19.65	11.45
						**HBoV**						

2015	0.58	0.70	0.68	5.18	9.05	8.18	2.46	0.18	0.00	0.56	0.23	0.55
2016	1.06	0.83	1.93	3.86	4.55	2.80	1.06	1.15	0.83	0.30	0.25	0.30
2017	0.58	1.00	1.35	4.66	9.33	3.98	0.82	0.30	0.25	0.18	0.20	0.58
2018	0.26	0.70	0.10	0.88	4.35	7.18	5.78	0.55	0.63	0.60	0.85	0.88
2019	0.52	0.58	1.40	1.78	4.88	11.78	8.22	2.00	2.05	1.16	1.20	1.10
**HMPV**												
2015	0.66	0.40	0.98	3.64	7.10	4.20	1.48	0.33	0.13	0.46	0.43	1.68
2016	3.56	4.20	8.05	12.64	8.53	2.83	0.66	1.20	0.98	0.42	1.05	1.14
2017	4.76	11.10	16.38	14.14	4.80	0.63	0.56	0.10	0.00	0.76	0.60	0.28
2018	0.86	2.95	8.60	19.12	15.33	6.00	2.82	0.73	0.48	0.08	0.18	0.15
2019	0.62	1.68	6.60	9.60	17.10	8.88	4.24	2.98	1.95	1.18	0.70	1.63
**Group A Rotavirus**												
2015	14.10	22.35	28.90	16.38	6.33	2.70	1.72	3.25	5.38	2.62	1.65	0.98
2016	5.66	16.45	21.08	13.28	6.03	3.73	2.30	4.20	3.43	1.86	1.68	2.76
2017	10.38	21.68	30.33	20.26	8.13	5.53	1.68	2.30	3.45	2.34	4.35	7.38
2018	14.52	19.53	16.88	10.10	4.38	4.55	1.84	1.98	0.85	1.90	3.13	4.10
2019	5.06	11.88	13.48	4.93	5.12	0.90	1.32	1.45	1.00	0.96	3.60	2.75
**Norovirus**												
2015	29.20	15.30	13.35	5.82	7.53	6.73	4.86	3.20	6.18	10.36	26.28	43.98
2016	38.62	21.85	20.33	13.64	7.80	4.08	3.66	3.40	2.65	9.32	28.58	41.06
2017	30.28	20.00	21.08	19.18	18.58	10.40	3.72	6.20	4.23	8.06	33.40	39.58
2018	25.46	19.20	10.78	15.74	9.30	9.90	4.34	3.20	5.35	6.28	14.95	16.90
2019	39.10	25.70	24.88	32.15	22.74	10.95	5.66	4.80	1.38	2.96	1.93	30.15
**Enteric Adenovirus**												
2015	1.68	3.03	1.55	1.70	2.30	1.05	0.28	0.00	0.60	2.22	0.55	1.80
2016	2.28	2.58	1.25	2.30	2.28	2.75	2.16	3.43	10.73	6.88	5.68	6.78
2017	2.10	2.23	0.55	2.86	2.90	3.05	2.78	3.30	3.50	5.04	3.43	1.13
2018	2.40	1.85	2.55	3.04	2.85	5.65	4.44	6.45	6.03	3.82	3.33	2.15
2019	0.00	0.85	2.08	2.25	2.00	2.18	1.26	4.08	1.43	1.00	0.80	1.28
**Astrovirus**												
2015	2.30	1.28	2.30	2.68	3.25	3.05	0.36	0.58	1.73	1.14	0.70	0.68
2016	0.94	1.18	2.48	3.20	3.40	4.30	2.52	2.28	3.43	2.82	2.05	0.86
2017	11.96	1.15	1.73	5.54	3.90	4.08	3.32	2.30	1.78	2.08	0.68	2.58
2018	0.94	1.73	0.00	1.82	3.05	2.50	2.74	5.18	5.63	3.98	2.73	2.33
2019	1.44	3.05	2.70	0.98	1.26	2.20	3.14	1.50	3.23	1.28	4.80	1.50

Table S2. The monthly incidence of febrile convulsion

	January	February	March	April	May	June	July	August	September	October	November	December	Total	Average
2015	1074	1338	1328	1607	1934	1031	1132	1346	973	812	959	1233	14767	1231
2016	1252	1684	1205	1481	1517	1588	1289	988	819	842	972	1574	15211	1268
2017	972	633	961	1341	1336	1246	1474	911	756	678	804	1249	12361	1030
2018	1263	763	731	1048	1228	1137	1115	726	680	642	793	1187	11313	943
2019	693	533	743	1087	1172	1181	1147	830	781	720	722	1030	10639	887
Total	5254	4951	4968	6564	7187	6183	6157	4801	4009	3694	4250	6273	64291	5358
Average	1051	990	994	1313	1437	1237	1231	960	802	739	850	1255	12858	1072

Figure S1: Positive detection rates of virus during study period

Mon / PDR(%)	Jan	Feb	Mar	Apr	May	Jun	Jul	Aug	Sep	Oct	Nov	Dec
**HAdV**												
2015	5.04	2.15	2.83	4.70	4.83	5.63	3.32	3.18	2.30	6.98	9.03	8.78
2016	9.18	3.88	3.93	5.54	6.53	6.95	7.16	9.48	9.58	6.50	5.83	3.24
2017	3.72	2.53	2.43	2.74	6.05	4.73	2.96	5.18	4.33	3.78	4.73	2.60
2018	2.98	2.80	4.25	5.38	5.45	6.80	5.82	9.55	13.33	11.30	10.70	7.63
2019	6.68	5.05	4.85	4.43	7.84	10.40	7.52	12.88	10.20	9.44	9.75	8.83
**HPIV**												
2015	0.92	0.55	0.78	6.30	17.65	11.75	7.04	6.28	8.93	4.72	9.53	4.08
2016	2.24	1.30	1.40	5.60	16.55	16.23	9.68	10.25	8.68	4.56	1.95	0.78
2017	1.58	1.70	2.98	8.38	19.43	12.83	9.68	8.95	4.60	3.04	3.33	2.68
2018	0.84	1.60	3.25	9.44	19.10	16.78	11.12	7.75	4.33	4.00	1.75	1.03
2019	1.06	1.10	2.33	3.48	13.56	20.35	15.74	9.70	7.58	3.98	2.55	1.65
**HRSV**												
2015	8.70	2.15	1.55	0.40	0.58	0.45	0.12	0.13	0.00	0.96	4.13	14.40
2016	8.56	1.00	1.43	0.56	0.70	0.15	0.34	0.28	1.78	6.46	17.83	13.02
2017	5.16	3.83	2.53	0.64	0.10	0.23	0.60	0.85	3.30	5.24	17.25	10.73
2018	4.58	4.25	3.65	0.64	0.50	0.40	0.72	1.05	1.55	4.52	13.95	11.20
2019	6.40	3.80	1.65	0.70	0.44	0.10	0.10	0.85	1.68	3.72	10.10	11.73
**IFV**												
2015	22.80	48.85	45.28	25.96	3.10	0.33	0.14	0.00	0.38	0.10	0.48	1.53
2016	15.24	47.55	36.95	26.60	5.00	0.70	0.22	0.28	0.25	0.40	0.80	35.50
2017	26.20	7.70	8.95	12.72	5.03	2.20	1.10	0.55	0.93	0.66	4.05	41.55
2018	58.50	35.63	10.55	3.46	2.80	1.08	0.50	0.30	0.58	1.78	11.15	35.35
2019	28.20	9.13	20.80	34.85	10.52	2.10	0.40	0.35	0.63	3.44	10.08	23.95
**HCoV**												
2015	8.74	1.40	0.95	0.56	0.10	0.50	0.58	0.60	0.38	1.38	1.80	5.53
2016	7.96	3.98	4.95	4.26	4.90	3.08	3.94	4.53	4.68	4.24	7.80	9.82
2017	10.26	6.45	5.15	3.62	2.68	2.35	1.02	1.45	1.55	2.14	5.15	7.20
2018	6.76	9.88	7.73	3.52	0.85	0.48	1.00	3.13	2.60	5.80	10.93	9.83
2019	5.48	3.70	2.83	1.38	0.92	1.68	0.64	0.45	1.05	1.94	3.35	7.55
**HRV**												
2015	8.80	7.73	10.08	15.84	17.10	19.30	21.62	18.10	25.40	28.82	29.03	16.78
2016	8.52	3.78	12.28	15.44	19.55	16.38	19.62	19.60	18.40	24.56	20.68	8.02
2017	9.56	15.40	20.90	18.66	14.78	17.78	21.40	30.63	33.10	24.68	23.88	6.83
2018	2.52	5.95	20.63	28.34	21.53	20.18	21.14	12.70	24.63	22.08	15.70	9.78
2019	8.68	14.33	18.85	16.43	19.82	17.45	23.00	13.45	21.48	22.62	19.65	11.45
**HBoV**												
2015	0.58	0.70	0.68	5.18	9.05	8.18	2.46	0.18	0.00	0.56	0.23	0.55
2016	1.06	0.83	1.93	3.86	4.55	2.80	1.06	1.15	0.83	0.30	0.25	0.30
2017	0.58	1.00	1.35	4.66	9.33	3.98	0.82	0.30	0.25	0.18	0.20	0.58
2018	0.26	0.70	0.10	0.88	4.35	7.18	5.78	0.55	0.63	0.60	0.85	0.88
2019	0.52	0.58	1.40	1.78	4.88	11.78	8.22	2.00	2.05	1.16	1.20	1.10
**HMPV**												
2015	0.66	0.40	0.98	3.64	7.10	4.20	1.48	0.33	0.13	0.46	0.43	1.68
2016	3.56	4.20	8.05	12.64	8.53	2.83	0.66	1.20	0.98	0.42	1.05	1.14
2017	4.76	11.10	16.38	14.14	4.80	0.63	0.56	0.10	0.00	0.76	0.60	0.28
2018	0.86	2.95	8.60	19.12	15.33	6.00	2.82	0.73	0.48	0.08	0.18	0.15
2019	0.62	1.68	6.60	9.60	17.10	8.88	4.24	2.98	1.95	1.18	0.70	1.63
**Group A Rotavirus**												
2015	14.10	22.35	28.90	16.38	6.33	2.70	1.72	3.25	5.38	2.62	1.65	0.98
2016	5.66	16.45	21.08	13.28	6.03	3.73	2.30	4.20	3.43	1.86	1.68	2.76
2017	10.38	21.68	30.33	20.26	8.13	5.53	1.68	2.30	3.45	2.34	4.35	7.38
2018	14.52	19.53	16.88	10.10	4.38	4.55	1.84	1.98	0.85	1.90	3.13	4.10
2019	5.06	11.88	13.48	4.93	5.12	0.90	1.32	1.45	1.00	0.96	3.60	2.75
**Norovirus**												
2015	29.20	15.30	13.35	5.82	7.53	6.73	4.86	3.20	6.18	10.36	26.28	43.98
2016	38.62	21.85	20.33	13.64	7.80	4.08	3.66	3.40	2.65	9.32	28.58	41.06
2017	30.28	20.00	21.08	19.18	18.58	10.40	3.72	6.20	4.23	8.06	33.40	39.58
2018	25.46	19.20	10.78	15.74	9.30	9.90	4.34	3.20	5.35	6.28	14.95	16.90
2019	39.10	25.70	24.88	32.15	22.74	10.95	5.66	4.80	1.38	2.96	1.93	30.15
**Enteric Adenovirus**												
2015	1.68	3.03	1.55	1.70	2.30	1.05	0.28	0.00	0.60	2.22	0.55	1.80
2016	2.28	2.58	1.25	2.30	2.28	2.75	2.16	3.43	10.73	6.88	5.68	6.78
2017	2.10	2.23	0.55	2.86	2.90	3.05	2.78	3.30	3.50	5.04	3.43	1.13
2018	2.40	1.85	2.55	3.04	2.85	5.65	4.44	6.45	6.03	3.82	3.33	2.15
2019	0.00	0.85	2.08	2.25	2.00	2.18	1.26	4.08	1.43	1.00	0.80	1.28
**Astrovirus**												
2015	2.30	1.28	2.30	2.68	3.25	3.05	0.36	0.58	1.73	1.14	0.70	0.68
2016	0.94	1.18	2.48	3.20	3.40	4.30	2.52	2.28	3.43	2.82	2.05	0.86
2017	11.96	1.15	1.73	5.54	3.90	4.08	3.32	2.30	1.78	2.08	0.68	2.58
2018	0.94	1.73	0.00	1.82	3.05	2.50	2.74	5.18	5.63	3.98	2.73	2.33
2019	1.44	3.05	2.70	0.98	1.26	2.20	3.14	1.50	3.23	1.28	4.80	1.50

Figure S2. Positive detection rates of virus during study period (plot).

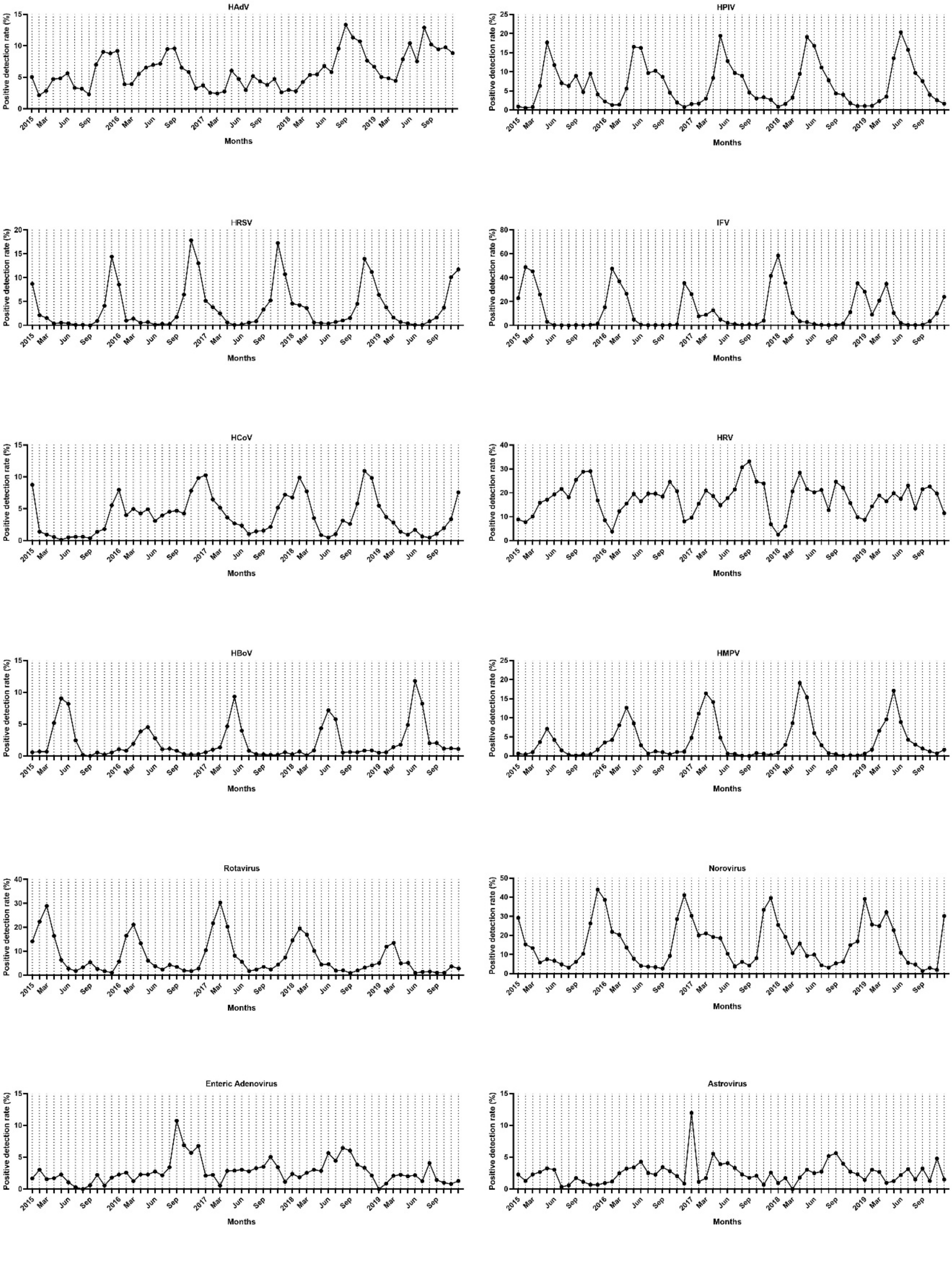

Supplement: Supplementary file 1 [file children-10-00529-s001.zip › children-2266394-supplementary.pdf]
